# Recruiting people facing social disadvantage: the experience of the Free Meds study

**DOI:** 10.1186/s12939-021-01483-6

**Published:** 2021-06-29

**Authors:** Pauline Norris, Kimberly Cousins, Marianna Churchward, Shirley Keown, Mariana Hudson, Leina Isno, Leilani Pereira, Jacques Klavs, Lucy Linqing Tang, Hanne Roberti, Alesha Smith

**Affiliations:** 1 Centre for Pacific Health, Va’a o Tautai, University of Otago, Dunedin, New Zealand; 2 Health Services Research Centre, Victoria University of Wellington, Wellington, New Zealand; 3 Turanga Health, Gisborne, New Zealand; 4 Kerry Nott Pharmacy, Opotiki, New Zealand; 5 School of Pharmacy, University of Otago, Dunedin, New Zealand

**Keywords:** Study recruitment, Social disadvantage, Health services research, New Zealand, Maori, Prescription charges

## Abstract

**Background:**

Researching access to health services, and ways to improve equity, frequently requires researchers to recruit people facing social disadvantage. Recruitment can be challenging, and there is limited high quality evidence to guide researchers. This paper describes experiences of recruiting 1068 participants facing social disadvantage for a randomised controlled trial of prescription charges, and provides evidence on the advantages and disadvantages of recruitment methods.

**Methods:**

Those living in areas of higher social deprivation, taking medicines for diabetes, taking anti-psychotic medicines, or with COPD were eligible to participate in the study. Several strategies were trialled to meet recruitment targets. We initially attempted to recruit participants in person, and then switched to a phone-based system, eventually utilising a market research company to deal with incoming calls. We used a range of strategies to publicise the study, including pamphlets in pharmacies and medical centres, media (especially local newspapers) and social media.

**Results:**

Enrolling people on the phone was cheaper on average than recruiting in person, but as we refined our approach over time, the cost of the latter dropped significantly. In person recruitment had many advantages, such as enhancing our understanding of potential participants’ concerns. Forty-nine percent of our participants are Māori, which we attribute to having Māori researchers on the team, recruiting in areas of high Māori population, team members’ existing links with Māori health providers, and engaging and working with Māori providers.

**Conclusions:**

Recruiting people facing social disadvantage requires careful planning and flexible recruitment strategies. Support from organisations trusted by potential participants is essential.

**Registration:**

The Free Meds study is registered with the Australian and New Zealand Clinical Trials Registry (ACTRN12618001486213).

## Background

There is a considerable amount of evidence that financial barriers reduce access to medicines, that they prevent vulnerable people from accessing medicines they need [1, 2], and that they may lead to poorer health [3] and increased need for healthcare [4–7]. However, there are very few randomised controlled trials (RCTs) comparing the health outcomes of people whose medications are provided free of charge with those where charges are applied. Our Free Meds study aimed to exempt some people from charges and compare their outcomes with those of people who continued to pay the standard charges to see if removing the charges might improve health outcomes. Recruiting participants for RCTs is often difficult and there is limited high quality evidence to guide researchers [8]. Many of the potential participants in our trial would be facing social disadvantage and marginalisation, so we anticipated recruitment might be particularly challenging [9]. Additionally, we wanted to ensure that we included substantial numbers of Māori (indigenous) and Pacific participants, because these groups are more likely to experience difficulties in paying for prescriptions [10] and as the INCLUDE framework makes clear, trials should involve those likely to be affected by the results [11]. Previous studies have found recruitment of Māori and Pacific people difficult [12, 13], although this is clearly possible if prioritised and adequately resourced [14]. The aim of this paper is to describe the process of recruitment for the Free Meds Study, what worked, what did not work, and the lessons we learnt in the recruitment process. The results of the study will eventually be reported elsewhere.

### The Free Meds study

In New Zealand almost all prescription medicines are funded by the government and incur only a standard $5 charge per item. Although this charge is low by international standards, about 5% of people report that they did not collect their medicines due to cost in the previous 12 months, and those living in the most deprived areas are six times more likely to report cost barriers as those living in the least deprived areas [15]. People in the Free Meds study were randomised to either the intervention group (who did not have to pay their $5 copayments for a year) or the control group (who continued as normal and then received a $100 grocery voucher at the end of the study year (i.e. Feb 2021)). Blinding of participants and recruiters was impossible but those recording outcome measures were blinded to whether people were in the intervention or control group.

The trial aimed to enrol people who were most likely to have problems paying for their medicines, and who may experience significant health problems and require hospital care if they stopped taking them. Therefore we decided to include people who were living in areas of high deprivation (using NZDep, an area-based measure of socioeconomic deprivation [16]) or were homeless, taking either medication for diabetes or antipsychotics (or both). People with diabetes often take several medications for diabetes and to reduce their risk of cardio-vascular disease, and often have other conditions [17]. People taking antipsychotic medicines often take several medications for physical as well as mental health problems [18, 19]. The study methods have been described elsewhere (Cousins K, Norris P, Horsburgh S, Smith A, Keown S, Samaranayaka A, et al: The impact of removing prescription charges on hospital bed-days and other health outcomes for people with low incomes and high health needs: study protocol for a randomised controlled trial, under review) but they are summarised here, along with eventual modifications to the planned design.

## Methods

### Study sites

We planned to carry out the study in three geographical areas: Tairāwhiti (a largely rural area with one small city, Gisborne), Dunedin, and Porirua. These were chosen to include rural and urban areas, large and small cities, and to provide ethnic diversity. We consulted with groups in these areas and were confident of their support in recruiting participants. These groups included mental health support providers, primary care providers, and organisations representing Māori and Pacific people. We had study team members based in Gisborne, Dunedin and close to Porirua, so we also planned to use our formal and informal networks for recruiting.

However, in July 2019, about 4 months before we intended to start recruitment, a large supermarket chain (Countdown) decided to waive $5 charges on all prescriptions dispensed in their onsite pharmacies. Therefore, we had to abandon plans to recruit in Dunedin and Porirua because the Countdown pharmacies would potentially supply people in the control group in those areas with free prescriptions, diluting any effect of the intervention. We had to make significant and hurried changes and move the study to smaller cities over a wider geographical area (avoiding the 12 other cities where Countdown had pharmacies).

Pharmacist cooperation was crucial to the study success. It was impossible to set up a centralised system where the intervention group could be flagged in a national dataset and receive free prescriptions, so we had to meet with all community pharmacists and ask them to invoice the study for prescriptions for those in the intervention group. When the new study areas were selected, we visited as many pharmacies as possible in these areas to tell them about the study and ask for their cooperation. Almost all were enthusiastic about the study, and were extremely helpful.

### Recruitment timeframe

The time for recruitment was very constrained because of the design of the prescription charges scheme in New Zealand. From 1 February each year, people pay for 20 prescription items and are then exempt from charges until the 31 January of the following year. Therefore, recruitment had to be completed by 1 February 2020 so that the intervention would have an impact for a full year. Recruiting too early was problematic since people were less likely to be concerned about prescription charges at that time of the year. Thus, we did our first recruitment event on 30 October 2019.

### Recruitment modes

Our initial plan was to recruit people in person. We planned to advertise where and when we would be recruiting, and set up a “stall” in a pharmacy, healthcare centre, or community venue and enrol people who came either deliberately to find us, or who happened to be visiting. In total, we did 43 of these recruitment events over 31 days. Venues included community centres, community pharmacies, medical centres and outside a general store in a rural area. In addition, we employed a Māori pharmacist in one of our areas who recruited patients at her pharmacy.

It quickly became clear that in person recruiting was expensive and often not very productive. Initially we obtained written informed consent, took multiple contact details and demographic information, randomised participants, and made a study ID card for those in the intervention group at our recruitment events. This required at least two staff members and usually three so that people could take breaks during long days. In the first month, we held 33 such events and the mean number of participants recruited per event was only 6.8 (range 1–37). It was clear that we could not rely on this model of recruiting.

After obtaining an amendment to our ethics approval, we introduced a phone system for enrolling participants. We obtained verbal consent on the phone but also attempted to obtain written consent by e-mail or text. We continued to advertise the study widely and those who were interested could call our free phone number. We answered this phone ourselves from 6 December to 15 January, including over the Christmas and New Year holiday and on occasional days after that (in total 40 days). In January, when numbers of participants was increasing, we employed a market research company, Infield International, who answered the phone, administered the recruitment questionnaire, and randomised participants.

We also expanded our inclusion criteria, so that people in slightly less deprived areas (NZDep7 as well as NZDep8–10) and people with chronic obstructive pulmonary disease (COPD) were also eligible. COPD was chosen because, as with the other conditions, people with COPD often take multiple medications and may need hospital care if they stop taking their medication [20].

We continued to recruit in person to meet the commitments we had already made, but this was much less frequent. After the first month, most in person recruiting was done by a sole researcher, and ID cards were made in our offices and posted to participants.

### Communication about the study

Throughout the recruitment period, we publicised the study by distributing pamphlets in pharmacies, GP practices and other venues, through media and Facebook. Local newspapers (such as the Wairoa Star, the Timaru Courier, the Levin Chronicle and Hawkes Bay Today) ran several stories about the project. We created a study Facebook page (Free Meds Study), made 62 posts on the page, and paid $500 in total for Facebook advertising (which Facebook refers to as “boosting” posts). People who saw the Facebook page still had to call our free phone number to enrol. We replied to all messages posted on our Facebook page or through Facebook Messenger. We tried to establish probable eligibility by Facebook Messenger before asking people to phone us. We were interviewed about the study on national and local radio stations, and attempted to contact local stations while recruiting in their area.

### Assessing effectiveness and costs of recruitment

We calculated the cost of recruitment per person recruited for each of the three recruitment strategies: in person, phone staffed by researchers, and phone staffed by the market research company. We excluded the cost of study set-up and visits to study areas to build relationships and liaise with health providers and community organisations, as these were necessary for all modes of recruitment. Staff salaries were included. While this was straightforward for research assistants paid by the hour, salaried staff worked well above the hours they were paid for, including weekends and holidays; however, only actual days for work and travel were included in the salary calculations. We did not include salary for a medical student who assisted in recruitment, funded by a summer studentship, but we did include her travel and other expenses.

## Results

Between 30 October 2019 and 7 February 2020, we recruited 1068 people to participate in the study. 357 (33%) were enrolled by the market research company and 711 (67%) by the research team. Of the latter, 474 (44% of the whole sample) were enrolled in person and the rest (237, or 22% of the whole sample) on the phone. Participant demographics are presented in Table 1.

**Table 1:** Participant demographics

		*n* (percent)
Gender		
Male		463 (43.4)
Female		605 (56.7)
Ethnicity*		
New Zealand European		541 (50.7)
Māori		520 (48.7)
Pacific		24 (2.2)
Other (including “Kiwi” and “New Zealander”)		107 (10.0)
Age		
Under 40		85 (8.0)
40–49		110 (10.3)
50–59		262 (24.5)
60–69		299 (28.0)
70–79		245 (22.9)
80 and over		67 (6.3)
Condition **		
Diabetes		729 (68.3)
Taking anti-psychotics (for any reason)		278 (26.0)
COPD		192 (18.0)
Sources of personal income Ɨ		
Wages, salary, commissions, bonuses		242 (22.7)
Self-employment		33 (3.1)
Interest, dividends, rent, other investments		35 (3.3)
Accident Compensation		12 (1.1)
Superannuation		474 (44.4)
Jobseeker benefit		82 (7.7)
Sole parent support		20 (1.9)
Supported Living payment		246 (23.0)
Other		38 (3.6)
No income		30 (2.8)

*Participants could report more than one ethnicity

**Usually, if people met one of the study criteria, we did not ask about others. Therefore, these numbers are likely to under-estimate the prevalence of the conditions. However, some recruiters did ask about all conditions, which is why the total is greater than the sample size

Ɨ Participants sometimes had more than one source of income.

During recruitment, we asked participants how they found out about the study, and the most common response was from a pharmacist (460 participants, or 43%). Health centres were also very significant sources of participants (224 participants). Facebook (137), flyers (109), family (90), and newspaper articles were also important (65). In total, 67 participants said they found out about the study by being approached in person by a member of the study team.

**Fig. 1:**
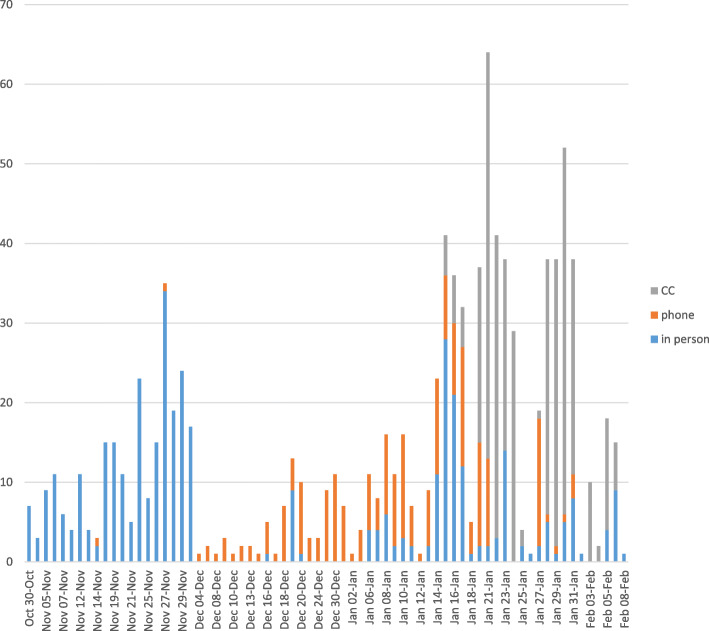
Numbers enrolled per day by method (call centre/phone/in person)

Numbers recruited per day tended to increase over time, up until 1 February. We continued to recruit in one area until 6 Feb, because we felt we had not provided enough communication in that region (Fig. 1).

**Fig. 2:**
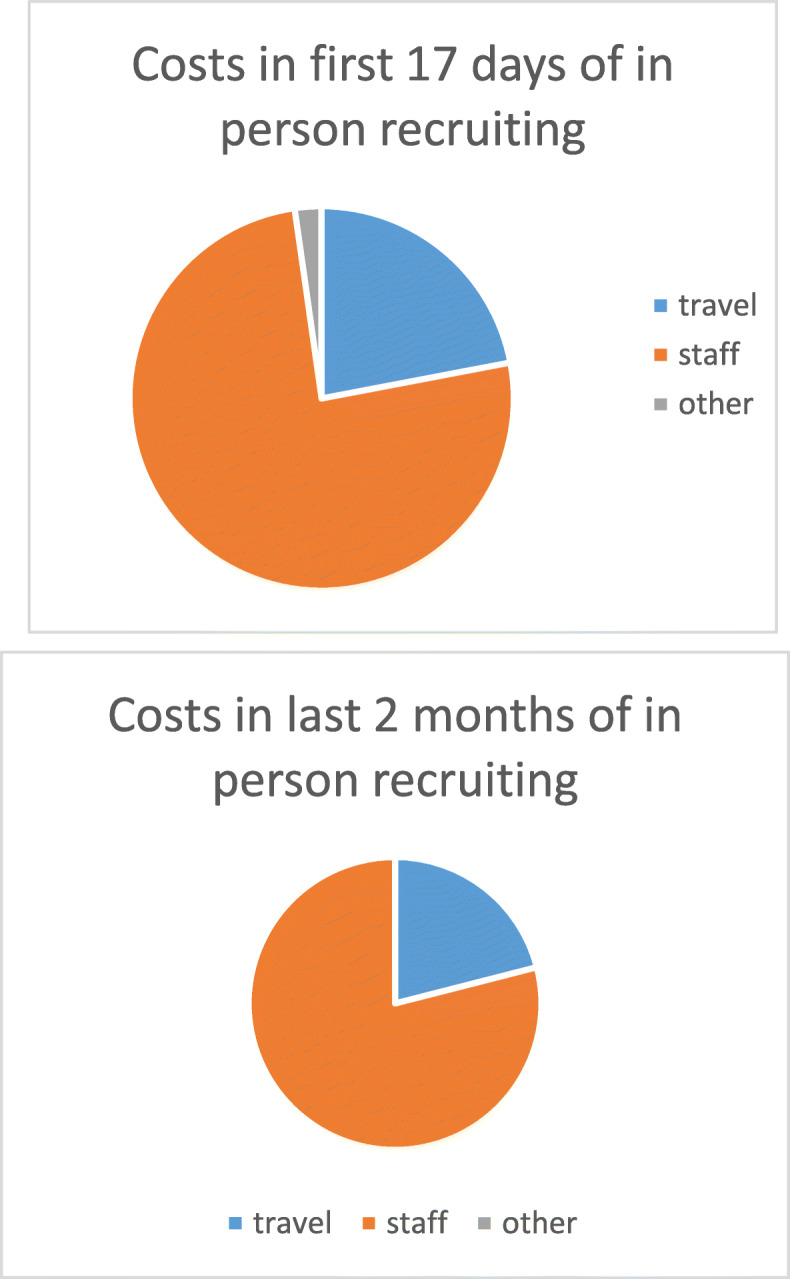
components of in person recruitment costs

Enrolling participants by phone was cheaper on average than enrolling them in person (Table 2). However, the success rate and cost of in person recruitment varied widely. In the first 17 days, where multiple team members were staffing a stall and numbers enrolled were very low, the average cost per participant was $203, whereas in the 2 months where we were much more selective in our approach and usually had only one staff member enrolling people, the average cost per participant was only $35 per participant. Salaries were consistently a high percentage of our costs (Fig. 2).

**Table 2:** Average cost per participant

Recruitment method	Average cost per participant (NZ$)
In person: Total	100.54 ($47,658 to recruit 474 people)
First 17 days	203.29 ($15,450 to recruit 76 people)
Final 2 months	34.82 ($8705 to recruit 250 people)
Phone with research team staffing the phone	77.40 ($18,345 to recruit 237 people)
Phone with call centre answering phone	54.90 ($19,602 to recruit 357 people)

## Discussion

We successfully enrolled a large number of people in a short period, averaging 356 per month. Many publications from trials do not provide enough data to determine recruitment rate but our rate is well above that reported in other studies. For example, in the UK, a review of trials funded and published by the Health Technology Assessment Programme found that trials recruited an average of less than one patient per centre per month [21]; and a review of US studies comparing virtual and traditional in person methods of recruitment found that virtual studies enrolled an average of 51 participants per month as compared to 13 participants per month among the more traditional studies [22]. In New Zealand, trials enrolling more than 1000 people have taken 9–13 months to recruit these [12, 13] and some with smaller numbers have taken much longer [23]. Our study required very little from participants and provided a direct financial benefit to them, so this is likely to have made recruitment much easier. However we faced similar challenges to others in informing people about the study and actually enrolling them. We were forced to try a range of methods to get enough participants for the study, which provided an opportunity to explore the strengths and weaknesses of each.

We enrolled roughly equal numbers of Māori and European (pākehā) participants. We attribute our success in recruiting Māori participants to having Māori researchers on the team, recruiting in areas of high Māori population, team members’ existing links with Māori health providers, and engaging and working with Māori providers [12, 14]. Despite our existing connections in Pacific communities, we failed to recruit substantial numbers of Pacific people. This is likely to be due to having to drop Porirua (which has a large Pacific population) as a study site, and the lack of time to develop relationships with Pacific organisations in other regions.

### Advertising the study

We found that local newspapers, especially free community newspapers, were the most useful media outlets. Radio stories and interviews did not seem to work, and it was hard to ensure that they included enough detail for people to contact us. We did not pay for advertising in traditional media, but found that in small towns it was very easy to get media attention simply by contacting reporters.

We used Facebook to advertise the study and, as others [13, 24] have reported, found it a very cheap and effective way to reach people. Although our Facebook page described the study as a University of Otago study, it was an independent page targeting a different audience to a University page [13]. Responding to posts and messages was not time consuming but was very disruptive to everyday life, especially because they were sent almost around the clock, and we felt it was important to respond quickly. Despite not being asked to, people often shared their medication lists and other very private information on the public Facebook page, which we felt very uncomfortable with. As non-digital natives we had a lot to learn about Facebook and wish we had had more skills starting out. Asking other organisations like pharmacies and patient groups to share our Facebook posts worked well but we found that people often commented and asked questions on these other pages, and we could not keep track of and respond to those comments and questions.

Distributing pamphlets, mainly through pharmacies, worked well. Initially we tried to make tailored pamphlets for each area, but this was complex and time-consuming, particularly preparing maps of eligible areas.

### Enrolling people in the study

Enrolling people in person was very labour intensive compared with phone. Our salary costs were high because senior staff participated in the recruitment. This allowed decisions to be made quickly and our approach to be adjusted as necessary, for example contacting local media and organisations, because there was no delay in contacting project leaders.

Initially in person enrolment was expensive but our approach became more targeted and efficient as we progressed. Some in person contact was essential, especially for informing people about the study, reminding people, and we think it helped considerably in attracting Māori participants. It was also useful for those who needed additional time for explanation or reassurance about confidentiality. A Cochrane review of qualitative studies on recruitment found that participants preferred in person explanations of studies because this provided the opportunity to ask questions and seek clarification [25] and others have found that in person approaches worked better for Māori [12]. In person recruiting at the start allowed us to meet some of our participants and potential participants so we learnt about their concerns about the study, which enabled us to make both our advertising and verbal explanations clearer.

We considerably over-estimated how many people we could reach through in person recruiting. Many of the people we worked with (community pharmacies, GP practices, clinics etc) also over-estimated how many eligible people would come in 1 day or to one session. In part, this seemed to be due to health professionals not taking the inclusion criteria seriously, thinking that we could enrol other people who did not fit the criteria, if they really needed free prescriptions. Some potential participants also seemed to interpret the criteria as indications of the seriousness of conditions or how much people deserved free prescriptions, so we sometimes had to reassure ineligible people that we sympathised with their situation, were not minimising its impact on their lives, but had to stick to the inclusion criteria for the study.

Recruiting worked best when we had a local advocate working with us, and when that advocate understood the inclusion criteria well. A practice nurse in one small rural practice knew all the locals, and rang them and asked them to come in while we were there. This was our busiest session. In another rural town, the pharmacy staff advertised the study on their Facebook page, rang people and asked them to come in, and told customers who came to pick up appropriate medication about the study and encouraged them to come to us in the corner of the store. In another instance, a Māori health provider asked kaiāwhina workers (support workers) to bring people in to enrol. On the other hand, another provider asked nurses to ring people and invite them to come in, but the nurses did not understand the criteria well, or did not have access to appropriate information, and so this did not work well.

Enrolling people by phone was much cheaper on average than in person and we found it was possible to generate rapport quite quickly on the phone. Using a phone system also gave us the option to outsource this work to a market research company, who provided an excellent service and enrolled about a third of our participants.

There were considerable collateral benefits of recruitment, beyond gaining study participants. Our principal funder (the Health Research Council of New Zealand) provided summer studentship funding for a Pacific medical student to work alongside the project, and this provided the student with experience of working in a research team, on a large project, and considerable learning about the health sector. We employed students as research assistants, and similarly they gained research experience, and one was subsequently employed by a pharmacy where he had recruited participants. The community pharmacist who we employed now has a research fellowship to continue to develop her research career. The research team valued the opportunity to network with healthcare providers, and one community pharmacist we met is now involved in another research project with us.

## Limitations

This paper outlines our experiences with recruiting, but we did not do any formal evaluation of the process, such as focus groups with stakeholders, employees or others involved in the process.

## Conclusion/recommendations

We successfully recruited more than 1000 people in socio-economically deprived areas of New Zealand in a very short timeframe. For others wishing to recruit participants for RCTs we recommend:

- Utilizing existing trusted relationships as much as possible. Community pharmacists were crucial for the success of our recruitment and many participants approached us saying ‘my pharmacist said I should do this’.
- Facebook is an excellent publicity tool. Facebook advertising is a cheap effective way to contact people, but the time involved in replying to messages should not be underestimated.
- As others have found, having Māori researchers on the team, working with Māori organisations, and in person recruitment work well for recruiting Māori participants.
- Choice of recruitment strategies and venues should be based on evidence as much as possible. For example, we should have asked clinics and pharmacies to look at their records and see how many people who were eligible for our study visited in a typical day. Similarly, we could have intercepted people in public places to determine how many we would have to approach to find people who were eligible. We wasted time and resources at the beginning of recruitment by spending time in places where there were few eligible people. We should have done in person recruiting only when requested by organisations who were committed to helping us and understood that we needed to adhere to the eligibility criteria.
- During recruitment, it is important to monitor methods, modify or abandon approaches that are not working, and develop new strategies quickly. Having project leaders directly involved in the recruiting facilitated this.

## Data Availability

The datasets used and/or analysed during the current study are available from the corresponding author on reasonable request.
